# Effects of medicinal plants mixture on growth performance, nutrient digestibility, blood profiles, and fecal microbiota in growing pigs

**DOI:** 10.14202/vetworld.2021.1894-1900

**Published:** 2021-07-24

**Authors:** Nguyen Cong Oanh, Truong Quang Lam, Nguyen Dinh Tien, Jean-Luc Hornick, Vu Dinh Ton

**Affiliations:** 1 Vietnam National University of Agriculture, Faculty of Animal Science, Ngo Xuan Quang Street, Trauquy, Gia Lam, 100000 Hanoi, Vietnam; 2 University of Liège, Faculty of Veterinary Medicine, FARAH Center, Department of Veterinary Management of Animal Resources, Quartier vallée 2, Avenue de Cureghem 6, B43a, 4000 Liège, Belgium; 3 Vietnam National University of Agriculture, Faculty of Veterinary Medicine, Key Laboratory for Veterinary Biotechnology, Ngo Xuan Quang Street, Trauquy, Gia Lam, 100000 Hanoi, Vietnam

**Keywords:** animal performance, blood profile, digestibility, growing pig, medicinal plants powder

## Abstract

**Background and Aim:**

Alternative natural materials to antibiotics for improving digestive health and growth performance are needed due to strengthening regulations related to the use of antibiotic growth promoters. The study aimed to evaluate the effects of medicinal plants mixture (60% *Bidens pilosa* L., 15% *Urena lobata* L., 15% *Pseuderanthemum palatiferum*, 5% *Ramulus cinnamomi*, and 5% *Star anise*) as alternative growth promotors on animal health, nutrient digestibility, blood parameters, and growth performance of growing pigs.

**Materials and Methods:**

The study was conducted, from April 2020 to June 2020, at a private pig production farm located in Cam Giang district Hai Duong Province, Vietnam. Forty-eight 10-week-old crossbred (♂Duroc×♀ [Landrace×Yorkshire]) pigs, average initial body weight 30.3±1.42 kg, were randomly allocated to four dietary groups, three replicate pens per experimental group, with 4 pigs/pen. For 7 weeks, the pigs were fed a basal diet supplemented with the mixture at levels of 0, 20, 40, and 60 g/kg of feed.

**Results:**

Final body weight, average daily gain, average daily feed intake, and feed conversion ratio, as well as apparent total tract digestibility of dry matter, organic matter, crude protein, ether extract, and gross energy were not significantly influenced by the diets (p>0.05). Inclusion of the plant mixture decreased significantly red blood cell count, blood cholesterol, urea nitrogen, and low-density lipoprotein (LDL) concentrations (p*<*0.05) compared with the control diet. No diet effect was observed on fecal *Escherichia coli*, *Salmonella* spp., *Clostridium* spp., and total bacteria counts.

**Conclusion:**

The incorporation of the plant mixture into the diet of growing pigs reduced serum cholesterol, LDL, and urea concentrations with no adverse effect on performance and nutrient digestibility.

## Introduction

Overuse of antibiotics for growth promotion and disease prevention in animals can contribute to the emergence of antibiotic resistance and increase human health risks [1]. Therefore, many countries have already taken action to reduce the use of antibiotics in food-producing animals. European Union has banned the use of antibiotics for growth promotion since 2006. Since then phytogenic compounds have been studied and used in animal feeds with the objectives to substitute antibiotics as growth promoters and disease prevention or treatment [2-5].

Several herbal extracts have been reported to promote feed intake, feed digestibility, and beneficial intestinal microbiome and also to improve immune system [6,7]. Medicinal plants *Ramulus cinnamomi* (*Cinnamon twig*), *Star anise* (*Illicium verum* Hook. f.), *Bidens pilosa* L., *Urena lobata* L., and *Pseuderanthemum palatiferum* are widely distributed in tropical and sub-tropical areas of Asia, especially Vietnam, and are considered as feed additives for animals [8]. The phytochemical components of these plants are tannins, saponins, phenols, alkaloids, and glycoside [9-13], which are known to be potential sources of useful drugs [14], especially exhibiting high antibacterial, immune, and antioxidant properties [12,15-18]. Flavonoids of *B. pilosa* protect the liver function by limiting an increase in hepatic levels of alanine aminotransferase (ALT) and aspartate aminotransferase (AST) on the carbon tetrachloride model in mice and no evidence of animal toxicity at 160 g/kg of animal live weight was observed [19]. A previous *in vitro* study [20] reported that water extract of *B*. *pilosa* had higher activity against *Bacillus cereus* and *Escherichia coli* than gentamycin sulfate. Dried and fresh leaves of *P. palatiferum* are used to treat diarrhea in piglets [21]. Recent *in vitro* studies [22,23] showed that *Star anise* and *R. cinnamomi* inhibited replication of influenza and flu virus. Moreover, weaning pigs fed a diet supplemented with 0.05% *S. anise* oil had a higher average daily gain (ADG) than those fed a control diet [24]. As far as we know, no studies reported the effects of a blend of more than 2 herbal plants of *B. pilosa, P. palatiferum, U. lobata, R. cinnamomi*, and *S. anise* on pig production.

The study aimed to evaluate the effects of medicinal plants mixture (60% *B. pilosa* L., 15% *U. lobata* L., 15% *P. palatiferum*, 5% *R. cinnamomi*, and 5% *S. anise*) as alternative growth promotors on animal health, nutrient digestibility, blood parameters, and growth performance of growing pigs.

## Materials and Methods

### Ethical approval

In the absence of proper regulation in Vietnam, all procedures included in the experiment were performed according to the best practices recommended by the ethical committee for experiments in animals of University of Liège, Belgium.

### Study period and location

The study was conducted from April 2020 to June 2020 at a private pig production farm located in Cam Giang district, Hai Duong Province, between latitude 20° 57’ 0” E and longitude 106° 13’ 0” E.

### Preparation of the medicinal plants

The plant species were chosen according to their availability around the farm and their constituents. Aerial parts of *P. palatiferum*, *U. lobata*, and *B. pilosa* were harvested during the vegetative growth phase at the medicinal garden of private agricultural farms located Cam Giang district, Hai Duong Province, Vietnam. *R. cinnamomi* and *S. anise* only were harvested during fructification from a forest garden at Huu Lung district, Lang Son Province, Vietnam.

The plants were dried separately in an oven at 60-75°C for 8 h. After drying, their materials were separately stored in air-tight bags. They were ground into fine powder and proportionally mixed as a powder (MP) containing 60% *B. pilosa*, 15% *U. lobata*, 15% *P. palatiferum*, 5% *R. cinnamomi*, and 5% *S. anise*. The weight contribution (60:15:15:5:5) of the medicinal plants in the mixture was determined by the availability of resources, their costs, and the recommendations of traditional medicine.

### Experimental design

Forty-eight crossbred (♂Duroc×♀ [Landrace×Yorkshire]) pigs, originating from eight sows, 3 to 4 litters order, initial body weight (IBW) 30.3±1.42 (SD), and age about 10 weeks old were used in this experiment. The pigs were individually ear tag numbered and randomly allocated to the four dietary treatments according to similar IBW, sex, and sow origin by treatment. There were three replicate pens per treatment, two barrows, and two gilts per pen (2 m×3 m) equipped with one stainless steel feeding and two automatic water drinking nipples. Animals were kept in a climate-controlled building with the temperature between 27 and 29°C, and the relative humidity between 70 and 85%.

Raw feed ingredients were purchased all at once from a local feed company. Feed was ground into flour through a 2 mm screen before the basal diet (T0) formulation. The experimental diets (T1, T2, and T3) were made of the basal diet mixed with MP at 20, 40, and 60 g/kg, respectively). Each was offered during a total experiment duration of 49 days. In the 2 last week of the experiment, chromium oxide (Cr_2_O_3_) was added at a level of 5 g/kg of diet, as an inert marker to measure digestibility parameters.

The complete diets were collected for chemical analysis. The basic ingredients and nutrient levels of the diets are shown in Table-1[25]. The nutrients composition of the diets met the recommended requirements for growing pigs [26]. Pigs were fed *ad libitum* during the whole experimental period and animals had free access to water by nipple drinkers.

**Table-1:** Ingredients and nutrient composition of the experimental diets.

Items	Dietary treatment^1^				MP

	T0	T1	T2	T3	
Ingredients (%, as-fed basis)					
Corn	33.9	33.2	32.6	31.9	
Soybean meal	13.4	13.1	12.8	12.5	
Fish meal	3.50	3.40	3.30	3.20	
Rice bran	25.0	24.5	24.0	23.5	
Wheat bran	20.0	19.6	19.2	18.8	
Limestone	1.50	1.50	1.40	1.40	
Vitamin-mineral premix^2^	0.50	0.50	0.50	0.50	
Salt	1.00	1.00	1.00	1.00	
Farm enzyme^3^	0.50	0.50	0.50	0.50	
L-lysine HCl, 98.5%	0.50	0.50	0.50	0.50	
DL-Methionine, 98%	0.20	0.20	0.20	0.20	
Medicinal plants powder^4^	-	2.0	4.0	6.0	
Analyzed composition (% DM) and energy value (MJ/kg DM)					
Dry matter	88.9	88.4	89.1	89.2	92.4
Crude protein	18.8	18.8	18.7	18.7	12.7
Ether extract	7.55	7.22	7.26	7.30	1.63
Ash	8.86	8.73	8.80	8.86	12.3
Crude fiber	5.24	5.89	5.97	6.95	28.4
Calcium	1.39	1.11	1.21	1.18	1.32
Total phosphorus	0.85	0.79	0.86	0.86	0.34
Gross energy (MJ/kg DM)	18.5	18.5	18.2	18.5	18.3
Metabolizable energy^5^ (MJ/kg DM)	13.9	13.8	13.7	13.5	-
Lysine^6^	1.33	1.32	1.31	1.30	-
Methionine^6^	0.54	0.53	0.53	0.53	-

MP=Medicinal plants power.

1 T0=Control diet; T2, T4, and T6: T0 supplemented with 2, 4, and 6% of MP, respectively. ^2^Premix in 1 kg: ZnSO_4_

(min-max): 250-300 mg; FeSO_4_ (min-max): 150-200 mg; MnSO_4_ (min-max): 150-200 mg; CuSO_4_

(min-max): 250-300 mg; Biotin: 8 mg; activity enzyme: 100 g; coarse sand (max): 2%; sufficient carrier for 1000 g; moisture (max): 10%. ^3^Enzyme in 1 kg: *Saccharomyces boulardii*: 10^9^-2.10^10^ CFU/g, *Saccharomycopsis fibuligera*: 10^6^-10^10^CFU/g, *Lactobacillus acidophilus*: 10^9^-3.10^9^ CFU/g, Candida tropicalis: 10^5^-10^8^ CFU/g, moisture (max): 10%. ^4^A blend powder of medicinal plants in 1 kg: 60%

*B. pilosa*, 15% U. lobata, 15% *P. palatiferum*, 5% *Ramulus cinnamomi*, and 5% *Star anise*. ^5^Calculated data according to the equation of [25] for ME

estimation: ME=4168-12.3×Ash+1.4×CP+4.1×EE- 6.1×CF (g/kg DM). ^6^Calculated data.

### Measurements

#### Animal performance

The animals were individually weighed at the start and the end of the experiment. Average feed intake, ADG, and feed conversion ratio (FCR) were calculated for each pen and diet treatment. In addition, disease symptoms such as diarrhea were recorded daily during the experiment.

#### Digestibility

For apparent total tract digestibility (ATTD), fresh fecal samples catched immediately after emission from all of the pigs were collected per pen 2 times/day (100 g/pig) for the past 5 days of the experiment and stored −20°C. At the end of the collection, the fecal samples were thawed, mixed, and pooled per pen, after which they were dried and analyzed for ATTD determination. The ATTD of nutrients was calculated using the following equation [27]:


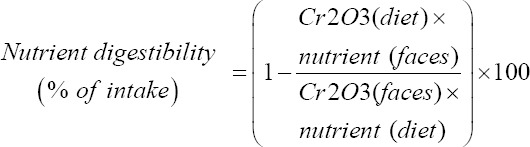


Where, nutrient digestibility is apparent digestibility of a nutrient or energy in the diet (%); nutrient (diet) is a nutrient (g) or gross energy (GE) (kcal) content per kg DM in diet and feces samples; and Cr_2_O_3_ (diet) and Cr_2_O_3_ (feces) are Cr_2_O_3_ content (g/kg DM) in diet and feces samples, respectively.

#### Blood and serum parameters

At the end of the experiment, 24 pigs (six pigs per treatment, one male and one female in each pen) were randomly chosen for blood sampling through the jugular vein using a sterile needle. The blood from each pig was collected into both K2EDTA (3 mL) and serum tubes (5 mL) (Zhejiang Gongdong Medical Technology Co. Ltd., Zhejiang, China). The blood samples in EDTA tubes were automatically analyzed using hematology analyzer ABX Pentra DX 120c to measure red blood cell (RBC) and white blood cell (WBC) counts, hemoglobin (Hb) concentration, and lymphocytes (WBC) percentage. The blood serum tubes were centrifuged at 3000 rpm for 15 min, and then, clean serum was collected to determine concentration levels of AST, ALT, bilirubin, total cholesterol, creatinine, high-density lipoprotein (HDL), low-density lipoprotein (LDL), protein, and urea using Cobas 8000 modular analyzer series (Roche-Hitachi, Tokyo, Japan).

#### Fecal microbiota

On day 28 of the experiment, fecal samples (about 50 g) of one male and one female per pen were collected directly from rectum, transferred to sterile falcons, and immediately placed on ice in an insulation box for a maximum of 1 h transportation to the laboratory for further analysis. At the laboratory level, 0.01 g of fresh fecal sample of each animal was taken and placed into Eppendorf tubes supplemented with 990 mL 1× phosphate-buffered saline and 6-fold dilutions were prepared. The samples were plated on agar plates. Bacteria were quantified based on colony-forming units (cfu) counting on the culturing plates (log10 cfu g^−1^). *E. coli* were enumerated on eosin methylene blue agar (Merck, Germany) after aerobic incubation at 37°C for 1 day. *Salmonella* species were enumerated on xylose lysine deoxycholate agar (Merck, Germany) after aerobic incubation at 37°C for 1 day. *Clostridium perfringens* were enumerated on Tryptose Sulfite Cycloserine agar (Merck, Germany) after anaerobic incubation at 37°C for 1 day. Total anaerobic bacteria were enumerated on nutrient agar (Merck, Germany) after anaerobic incubation at 37°C for 1-2 days. Before statistical analysis, fecal microbiota concentrations were transformed (log).

### Chemical analysis

Diets and feces samples were pre-dried by oven drying at 65°C for 72 h and then milled separately through a 1 mm screen before analysis. Diets and feces were analyzed for crude protein (CP); method 954.01 [28], ether extract (EE) with petroleum ether solvent (method 920.39 [28]), ash (method 942.05 [28]), crude fiber (CF; method 962.09 [28], neutral detergent fiber (NDF) with fiber filter bags of Ankom Technology F57 (method 973.18 [28]), GE through bomb calorimeter E2K (Germany), and chromium concentration was analyzed using ultraviolet absorption spectrophotometry (DR 3000, Germany) as previously described [29].

### Statistical analysis

All data were analyzed using the PROC MIXED procedure of SAS (version 9.4, Institute Inc., Cary, NC, USA), and each pen was used as an experimental unit. The statistical model included the diets (n=4) as a fixed effect and the blocks (n=3) as random effects. Pen was used as an experimental unit for growth performance and digestibility data, and individual pig as an experimental unit for fecal microbiota, and blood profiles. For repeated measures performed on the same experimental unit, a similar model was used but including the effect of a compound symmetry structure of covariance. Orthogonal polynomials were performed to determine linear and quadratic effects of increasing levels of MP in diets [30]. Data for pigs fed diets containing MP were compared with data for pigs fed control diet using orthogonal contrasts. The multiple comparisons of least-square means were performed according to Tukey adjustment. Significance was defined as p*<*0.05 and 0.05*<*p*<*0.10 were considered as a trend.

## Results

### Animal performance, health, and digestibility

The performances of the experimental pigs are shown in Table-2. There were no animal losses during the experimental period in the present study and no episodes of diarrhea or other symptoms were observed in the different groups. The IBW was not significantly different between dietary treatments and there were no significant effects between groups on FBW, ADFI, and FCR (Table-2). Effects of increasing levels of MP on ATTD are presented in Table-3. No significant differences in the ATTD of DM, OM, CP, EE, and GE were observed between diet groups.

**Table-2:** Feed intake, average daily gain, and feed conversion ratio (LSM) of growing pigs fed diets containing different levels of medicinal plants powder.

Items	Dietary treatment^1^				SEM	p-value	
	
	T0	T1	T2	T3		Linear	Quadratic
n	12	12	12	12			
IBW (kg)	30.3	30.4	30.3	30.3	0.42	0.93	1.00
FBW (kg)	65.6	65.1	64.6	64.2	1.49	0.48	0.98
ADFI (kg/d)	1.84	1.86	1.83	1.80	0.04	0.37	0.56
ADG (g/d)	750	737	731	720	31.8	0.50	0.98
FCR (kg/kg)	2.46	2.53	2.51	2.50	0.06	0.64	0.53

LSM=Least squares mean; SEM=Standard error of the mean; n=Number of animals; d=Day; g= Gram; IBW=Initial body weight; FBW=Final body weight; ADG=Average daily gain; ADFI=Average daily feed intake; FCR=Feed conversion ratio (kg feed/kg gain).

1 T0=Control diet; T1, T2, and T3 supplemented with 2, 4, and 6% of blend powder of medicinal plants.

**Table-3:** Apparent total tract digestibility (LSM) of growing pigs fed diets containing different levels of medicinal plants powder (%).

Items	Dietary treatment^1^				SEM	p-value	
	
	T0	T1	T2	T3		Linear	Quadratic
DM	92.49	92.61	91.56	92.44	0.40	0.79	0.90
OM	79.15	78.29	77.91	77.20	1.01	0.69	0.28
CP	80.88	79.71	78.68	78.43	1.07	0.51	0.14
EE	72.51	70.02	70.47	71.52	2.49	0.47	0.92
GE	77.99	75.46	76.90	76.29	1.19	0.22	0.92

LSM=Least squares mean; SEM=Standard error of the mean.

1 T0, control diet; T1, T2, and T3 supplemented with 2, 4, and 6% of blend powder of medicinal plants. DM=Dry matter; OM=Organic matter; CP=Crude protein; EE=Ether extract; GE=Gross energy.

### Blood characteristics

Significant linear decreases in RBC (p=0.003), and plasma cholesterol, LDL, and urea nitrogen (p*≤*0.04) were observed in pigs fed MP diets compared with those fed the control diet. A trend for a linear decrease in lymphocytes proportions (p=0.05) and a trend for open-up quadratic effect (p*≤*0.08) on creatinine and total protein concentrations were observed between diet groups. The other blood parameters including WBC, Hb, AST, ALT, bilirubin, or HDL were unaffected by the dietary treatments (Table-4). Besides, using orthogonal contrasts, decrease in RBC (p*<*0.05), decrease trend in plasma cholesterol, and LDL (p*≤*0.09) were observed when animals fed MP diets were compared with the control animals.

**Table-4:** Blood parameters (LSM) of growing pigs fed diets containing different levels of medicinal plant powders.

Items	Dietary treatment^1^				SEM	p-value	
	
	T0	T1	T2	T3		Linear	Quadratic
Number of animals	6	6	6	6		
WBC (G/L)	27.0	24.4	22.0	26.7	2.28	0.76	0.15
RBC (T/L)	7.60	6.92	6.92	6.62	0.16	0.003	0.26
Hb (g/dL)	12.4	11.9	11.5	11.2	0.59	0.17	0.85
LYM (%)	69.5	58.8	60.6	52.0	4.97	0.05	0.84
AST (U/L)	37.7	57.9	37.9	43.1	8.63	0.92	0.40
ALT (U/L)	51.7	61.2	54.0	65.7	4.61	0.12	0.82
Bilirubin (µmol/L)	1.75	0.50	0.60	0.75	0.58	0.28	0.25
Creatinine (µmol/L)	137	117	118	134	9.63	0.88	0.08
Total cholesterol (mmol/L)	2.37	2.16	2.06	1.91	0.10	0.01	0.77
HDL (mmol/L)	0.97	1.04	0.99	0.98	0.03	0.97	0.22
LDL (mmol/L)	1.09	0.98	0.86	0.77	0.08	0.02	0.92
Total protein (g/L)	70.4	66.0	66.7	68.5	1.42	0.44	0.05
Urea nitrogen (mmol/L)	3.91	3.90	3.13	2.90	0.37	0.04	0.77

LSM=Least squares mean; SEM=Standard error of the mean; WBC=White blood cell count; RBC=Red blood cell count; Hb=Hemoglobin; LYM=

Lymphocyte; AST=Aspartate aminotransferase; ALT=Alanine aminotransferase; HDL=High-density lipoprotein; LDL=Low-density lipoprotein.

1 T0, control diet; T1, T2, and T3 supplemented with 2, 4, and 6% of blend powder of medicinal plants.

### Fecal microbiota

Fecal *E. coli*, *Salmonella*, *Clostridium*, and total bacteria concentrations were not significantly altered (p>0.05) by the treatments (Table-5).

**Table-5:** Fecal microbial shedding (LSM) of growing pigs fed diets containing different levels of medicinal plant powders (log CFU/g of wet feces).

Items	Dietary treatment^1^				SEM	p-value	
	
	T0	T1	T2	T3		Linear	Quadratic
Number of animals	6	6	6	6			
*Escherichia coli*	6.15	6.14	6.07	6.19	0.20	0.97	0.76
*Salmonella*	2.13	2.05	2.15	4.13	0.95	0.98	0.97
*Clostridium*	1.30	0.00	1.78	0.00	0.58	0.22	0.63
Total bacteria	8.10	8.31	7.85	7.94	0.25	0.30	0.63

LSM=Least squares mean; SEM=Standard error of the mean.

1 T0, control diet; T1, T2, and T3 supplemented with 2, 4, and 6% of blend powder of medicinal plants.

## Discussion

In the present study, feeding increasing levels of MP to growing pigs had no significant effect on FBW, ADG, ADFI, and FCR. In agreement with our results, Hanczakowska *et al*. [31] reported that a diet containing herbal extract mixture of *Salvia officinalis*, *Urtica dioica*, *Magnolia officinalis*, and *Echinacea purpurea* unaffected the growth performance of pigs when compared to those fed a control diet. Ahmed *et al*. [32] reported that growing-finishing pigs fed a diet containing medicinal plants (pomegranate, *Ginkgo biloba*, and licorice) showed no changes in live body weight and ADG compared to a control group. Utiyama *et al*. [33] reported a similar ADG in weanling pigs fed a diet with medicinal extract (garlic, clove, cinnamon, pepper, thyme, cinnamaldehyde, and eugenol) compared to a control group. However, Yan *et al*. [34] demonstrated that growing pigs fed a diet supplemented with the inclusion of medicinal extract (buckwheat, thyme, curcuma, black pepper, and ginger) had improved ADG and ADFI but not FCR. Marcin *et al*. [35] reported that piglets fed a diet supplemented with extracts of sage and oregano also had significantly improved ADG. The world of phytochemistry being vast, the contradictory results regarding growth performance responses to natural, dried, or extracted herbs may be easily ascribed to different species, plant parts and their physical properties, age of plant, harvest time, processing method, and different dosage used [6,36,37]. Secondarily, different physiological periods of animal and housing conditions may respond differently to herb supplementation [6,31]. ATTD of nutrients was similar in pigs fed MP diets compared with those fed the control diet during 7 experimental weeks. Similar results were observed in the previous experiment [38] who reported that nutrient digestibility of growing pigs fed diet with herbal flavor supplementation for 10 experimental weeks was not affected. This suggests that the digestibility of moderate amounts of herbal plants could be similar to that of a control diet.

Dietary supplementation with medicinal plants may have a beneficial effect on hematological and biochemical characteristics of pigs. Supplementation with fermented medicinal plants (*Gynura* *procumbens*, *Rehmannia glutinosa*, and *Scutellaria baicalensis*) increased WBC concentration in growing pigs [39]. Increase in RBC counts and blood lymphocytes concentrations was found in weaning pigs fed a diet supplemented with fermented garlic powder at 0.5, 1, and 2 g/kg feed [40]. Diets supplemented with herb extract mixture (buckwheat, thyme, curcuma, black pepper, and ginger) at 250 mg and 500 mg/kg feed increased significantly WBC and RBC counts and blood lymphocytes concentrations compared with the control diet at end of the experiment [34]. In the current work, no significant differences on WBC, Hb, and lymphocytes – but a trend – concentrations were observed in pigs fed MP diets compared with those fed the control diet. However, a significant decrease in RBC count was observed in pigs fed MP diet compared to pigs fed the control diet. This could be due to the probable presence of high polyphenol content in MP which is known to cause hemolysis of RBC [41]. Despite this decreased RBC count with inclusion of MP, hematological indicators were found to be within normal ranges for growing pigs, as reported previously [42,43]. On the other hand, pigs fed MP diet had significantly lower total cholesterol, LDL, and urea nitrogen than those from the control diet. Plant polyphenols markedly enhance cholesterol secretion into bile [44], which, in conjunction with the unabsorbed cholesterol, results in increased fecal excretion of cholesterol without affecting the excretion of bile acids, and plant polyphenols intake lowers, therefore, plasma cholesterol levels in animals. Dietary supplementation of an herbal plants combination in natural form (pomegranate, *G. biloba*, and licorice) decreased serum cholesterol level [32]. Thus, in our study, a decrease in serum total cholesterol level can be due to the probable presence of polyphenol in MP diets. On the other hand, flavonoids, which are reported to be present in the medicinal plant presently used, reduced cholesterol biosynthesis due to inhibition hepatic 3-hydroxy-3-methyl-glutaryl-CoA reductase and acyl CoA: cholesterol O-acyltransferase [45]. Lower plasma cholesterol levels observed in the present experiment could thus be due to the conjunction of the two above phenomenon. In addition, lower serum urea content was observed in pigs fed MP diets compared with those fed the control diet. A previous study [34] reported that growing pigs fed diet with herbal plants decreased NH_3_ and H_2_S concentrations of feces compared with those fed the control diet. Thus, MP could have promoted urea translocation from blood to intestine lumen, especially due to higher crude fiber content in the experimental diets. However, this hypothesis must be confirmed.

In our study, MP diets did not inhibit in pig feces the counts of the pathogenic bacteria studied compared with the control diet, which is consistent with a variety of medicinal plants as reported previously [46] who indicated that herbal plants did not inhibit the growth of *E. coli* and even enhance bacteria growth in gut intestinal of animals. Similarly, no significant differences in intestinal microbiology and diarrhea occurrence were observed in weanling pigs fed a diet with herbal extract mixture (garlic, clove, cinnamon, pepper, thyme, cinnamaldehyde, and eugenol) compared to those fed a control diet [33]. The present results indicate that the plant mixture did not show any bacteria modulating effect in gut on growing pigs.

## Conclusion

From the present study, it can be concluded that growing pig diets containing up to 60 g/kg of a blended power of medicinal plants (*B. pilosa*, *U. lobata*, *P. palatiferum*, *R. cinnamomi*, and *Star anise*) reduced blood cholesterol, LDL, and urea nitrogen concentrations without influencing performance, nutrient digestibility, and fecal microbiota of growing pigs. A limitation of this study is lacking interaction tests among medicinal plants. Further studies should be performed to obtain more comprehensive results.

## Authors’ Contributions

NCO, VDT, and HJ: Conceived and designed research. NCO and NDT: Conducted the experiment and collected samples. NCO and TQL: Analyzed the samples. NCO and HJ: Analyzed the data. NCO: Wrote original draft. All authors read and approved the final manuscript.
